# Keeping the Teeth Clean

**Published:** 1876-11

**Authors:** 


### KEEPING THE TEETH CLEAN.

At a meeting of the American Academy, Dec. last, a paper was read by Dr. H. I. Bowditch, on the animal and vegetable parasites infesting the Teeth, with the effects of different agents in causing their removal and destruction. Microscopical examinations had been made of the matter on the teeth and gums of more than forty individuals, selected from all classes of society, in every variety of bodily condition; and in nearly every case animal and vegetable parasites in great numbers had been discovered.—Of the animal parasites there were three or four species, and of the vegetable, one or two. In fact, the only persons whose mouths were found to be completely free from them, cleansed their teeth four times daily, using soap once. One or two of these individuals also passed a thread between the teeth to cleanse them more effectually. In all cases the number of the parasites was greater in proportion to the neglect of cleanliness. The effect of the application of various agents was also noticed. Tobacco juice and smoke did not impair their vitality in the least. The same was also true of the chlorine tooth-wash, of pulverized bark, of soda, ammonia, and various other popular detergents. The application of soap, destroys them.

THE condition of the human system most liable to terminate in some incurable disease, is that of habitual "constipation." And still a majority of persons not actively employed suffer from it. It should be known to everybody that "Nelaton's Suppository" will radically cure ninety-nine cases in a hundred. Where costiveness depends upon "piles," the cure is certain; no case of piles will resist this treatment for two days. Price 50 cents a box. Sent free on receipt of price. HALL & RUCKEL, 218 Greenwich Street, New York.

